# Flexural Beams as Mechanical Fabry–Perot Resonators: A Theoretical Framework for Dispersive Waveguide-Based Sensing

**DOI:** 10.3390/s26092622

**Published:** 2026-04-23

**Authors:** Mostafa Rahimi Dizadji, Songwei Wang, Vahid Jafarpour, David Adrian Reynoso, Haiying Huang

**Affiliations:** Department of Mechanical and Aerospace Engineering, University of Texas at Arlington, Arlington, TX 76010, USAsongwei.wang@mavs.uta.edu (S.W.); vxj0203@mavs.uta.edu (V.J.); dxr7257@mavs.uta.edu (D.A.R.)

**Keywords:** Fabry–Perot resonator (FPR), flexural beam, dispersive wave, waveguide-based sensors, fringe analysis, time–frequency analysis, group velocity

## Abstract

Fabry–Perot resonator (FPR) sensors are widely implemented in optical and microwave waveguides because their interference fringe spectra enable highly sensitive, stable, and calibration-free measurements. In contrast, despite the extensive use of beams and plates as waveguides in vibration- and ultrasound-based structural health monitoring (SHM), an explicit FPR framework for these mechanical waveguides has not been established. This paper demonstrates that flexural beams can be rigorously treated as FPRs despite their inherently dispersive nature. Through analytical derivation, wave-propagation analysis, and fringe-based group-velocity extraction, we show that flexural-beam resonances arise from multi-reflection interference analogous to Fabry–Perot interference. A closed-form relationship between the frequency-dependent group velocity and the FPR free spectral range (FSR) is established, enabling inverse determination of mechanical or environmental perturbance from the FPR fringe spectrum. By extending FPR-based fringe analysis to dispersive mechanical waveguides, this work introduces a theoretical framework for implementing dispersive mechanical waveguide-based FPR sensors.

## 1. Introduction

Fabry–Perot resonators (FPRs) are fundamental to modern optical sensing because they convert minute physical or environmental perturbations into measurable shifts in interference fringe spectra [1,2,3]. A Fabry–Perot (FP) cavity consists of two partially reflective mirrors that confine optical waves, producing multiple reflections that interfere constructively or destructively depending on the effective cavity length and frequency [4]. Small variations in temperature, pressure, strain, or refractive index modify the effective cavity length and shift the interference fringe frequencies [3]. Due to multiple reflections, these small changes translate into detectable fringe frequency shifts. By computing the effective cavity length from the free spectral range (FSR), namely the spectral separation between adjacent fringes, FPR measurements are inherently calibration-free and robust against light intensity fluctuations, connector losses, or long-term drift [5,6]. As a result, FPR-based fringe analysis enables highly repeatable, stable, and ultra-sensitive estimation of a large range of measurands, leading to widespread adoption of FPRs in high-resolution spectroscopy [7], environmental monitoring [8], biomedical diagnostics [9], and industrial applications [10].

Optical fiber FPR sensors, particularly intrinsic Fabry–Perot interferometric (IFPI) sensors, have been extensively studied for structural health monitoring (SHM) [11]. Multiple IFPI sensors can be fabricated along a single strand of optical fiber by etching short air gaps or locally modifying the fiber core to create partially reflective surfaces [12]. This configuration enables compact, stable, and highly sensitive sensors that support wavelength-multiplexed and distributed sensing along a single fiber. Using optical fibers as waveguides, IFPI optical fiber sensors can operate reliably in extreme temperatures, environments with strong electromagnetic interference, and remote locations where electronic sensors fail [13]. More recently, the IFPI sensor concept has been extended to the microwave domain by machining impedance discontinuities that act as partially reflective “mirrors” within the coaxial cables [14]. These discontinuities form FP cavities that generate interferometric fringe patterns shifting with changes in cavity length or the surrounding medium’s permittivity [15]. The successful extension of fundamental FPR concepts from optical fiber to microwave coaxial cables establishes FPR theory as a general wave-interference framework for implementing waveguide-based sensors.

Structural components such as bars, beams, and plates also function as waveguides that guide stress waves over long distances with relatively low attenuation [16,17]. These guided mechanical waves are highly sensitive to geometric or material perturbations, forming the basis of vibration- or ultrasound-based SHM [18,19]. In current practice, resonances in mechanical waveguides are primarily interpreted using modal analysis [20]. Although modal analysis accurately predicts resonance frequencies and mode shapes, it does not provide an interference-based spectral framework analogous to FPR. Consequently, the resonance characteristics of mechanical waveguides are rarely analyzed in terms of wave propagation and interference [21]. Similarly, guided-wave-based SHM focuses on time-of-flight, attenuation, or resonant frequency shifts rather than on multi-reflection interference or fringe spectra [19,22]. Despite extensive research on vibration- and ultrasound-based SHM, an explicit connection between FPR and mechanical waveguides, especially dispersive ones, remains largely unexplored.

Recent work demonstrated that lossless longitudinal bars behave as FPRs, thereby extending the FPR concept to non-dispersive mechanical waveguides [23]. Extending this framework to flexural beams, however, is nontrivial because classical optical FPR theory assumes non-dispersive wave propagation, whereas flexural waves in beams are dispersive; i.e., their phase and group velocities are frequency-dependent [16]. Although several prior studies have recognized that flexural-beam resonances originate from wave interference and satisfy phase-closure conditions consistent with FPR phase-matching conditions [24,25,26], these studies remain confined within classical vibration theory. Without recognizing the analog between flexural beams and FPR, these works did not explore FPR fringe analysis, which is the foundation for FPR-based sensors, to analyze the resonance characteristics of flexural beams. Similarly, acoustics and structural dynamics studies interpreted acoustic and transmission-line resonators in terms of multiple reflections and impedance mismatch [16,17,27,28]. To the best knowledge of the authors, no published work has investigated the application of FPR concepts for analyzing the resonant spectra of these acoustic resonators. The authors’ group has recently produced convincing experimental results showing that extending the FPR fringe analysis to longitudinal bars and flexural beams enables reliable detection of attenuation parameters [29,30]. However, unlike the published work proving that non-dispersive longitudinal bars are FPRs [23], no theoretical framework has been published that provides rigorous proof and analysis of dispersive flexural beams as FPRs.

The present work explicitly casts the frequency response function (FRF) of flexural beams with generic boundary conditions into the generic FPR Airy function and thus demonstrates that flexural beams are FPRs. Time–frequency analysis (TFA) of the flexural-beam FRF further reveals the FPR nature of wave propagation within flexural beams. By deriving a closed-form relationship between the FSR and the frequency-dependent group velocity, this work demonstrates that FPR fringe analysis concepts can be applied to dispersive mechanical waveguides and thus establishes a theoretical framework for implementing FPR sensors in dispersive mechanical waveguides.

## 2. Airy Function of Optical Fabry–Perot Resonator

The theoretical foundation of optical FPRs is well-documented [1,2]. This section summarizes the generic Airy distribution of optical FPR reported by [4] and the fundamental physical insights derived from it.

**Figure 1:**
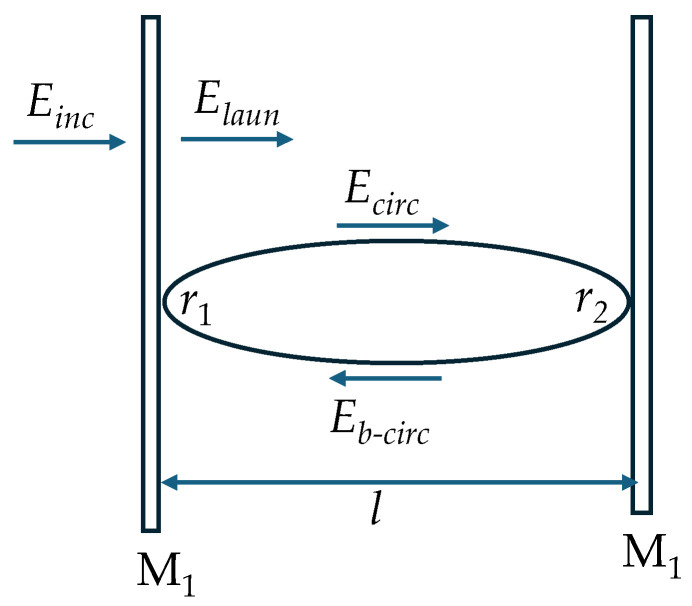
Optical Fabry–Perot resonator formed by two mirrors M_1_ and M_2_.

As illustrated in Figure 1, an optical FPR consists of two parallel mirrors M_1_ and M_2_ separated by a distance *l*. The left mirror is partially transparent allowing light to enter the space between the two mirrors, i.e., the FP cavity, while the right mirror can be partially transparent or completely reflective. The light launched into the FP cavity is labeled *E*_laun_, which is reflected by the right mirror and propagates toward the left as *E*_b-circ_. Reflected again by the left mirror, it propagates toward the right mirror as *E*_RT_. The forward-propagating light wave *E*_circ_ can be described by a generic Airy function as [4]

(1) $$E_{circ}=\frac{E_{laun}}{1-r_{1}r_{2}e^{-i\varphi }},$$

in which *r*_1_ and *r*_2_ are the reflections at the two mirrors. For an FPR with a lossy medium, the phase shift *φ’* is complex, i.e., *φ’= φ_r_ + iφ**_i_***. As such, Equation (1) can be rewritten as

(2) $$E_{circ}=\frac{E_{laun}}{1-r_{1}r_{2}e^{\varphi_{i}}e^{-i\varphi_{r}}}=\frac{E_{laun}}{1-re^{-i\varphi_{r}}}.$$

The total FPR optical loss, i.e., *r = r*_1_*r*_2_$e^{\varphi_{i}}$, includes the reflections at the two mirrors, i.e., *r*_1_ and *r*_2_, and the propagation loss $e^{\varphi_{i}}$. For most optical FPRs, the propagation loss is small and thus $e^{\varphi_{i}}$ can be neglected. The real part of the phase shift *φ_r_* is determined by the effective propagation path and the wave frequency.

Assuming *r*$e^{-i\varphi_{r}}$ is a small value, expanding Equation (2) using the Taylor expansion results in

(3) $$E_{circ}=E_{laun}\sum_{m=0}^{\infty }{\left(re^{-i\varphi_{r}}\right)}^{m}=E_{laun}\left(1+re^{-i\varphi_{r}}+r^{2}e^{-i2\varphi_{r}}+\ldots \right).$$

Equation (3) reveals that *E*_circ_ is a superposition of light waves launched into the cavity and those traveling multiple round trips between the two mirrors, providing a more physics-based interpretation of the Airy function. During each round trip, the amplitude of the light wave is attenuated by *r* and its phase is shifted by *φ_r_.*

The intensity of the circulating light wave *I_circ_* is calculated as

(4) $$I_{circ}=E_{circ}E_{circ}^{*}=\frac{I_{laun}}{1+r^{2}-2rcos\left(\varphi_{r}\right)}.$$

**Figure 2:**
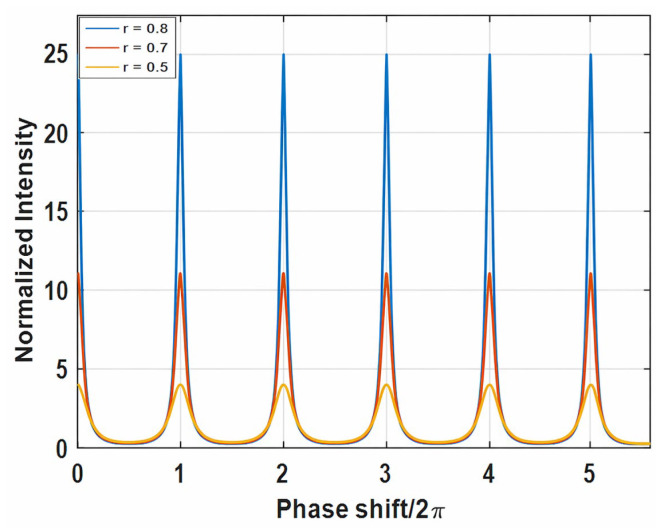
Normalized fringe spectrum of Fabry–Perot resonator with different FPR loss r.

Plotting the normalized intensity, i.e., *I_circ_/I_laun_*, versus the phase shift *φ_r_*/2*π* produces the FPR fringe spectrum shown in Figure 2. The peaks, called fringes in FPR terminology, reach a maximum when

(5) $$\varphi_{r}=2m\pi ,\;m=0,\;1,\;2,\ldots \;.$$

Physically, this means that the terms in Equation (3) have matching phases and interfere constructively at the fringe frequencies. As such, Equation (5) is called the FPR phase-matching condition. The FPR loss r determines the finesse of the fringes, i.e., the sharpness of the fringe peaks, but does not influence the fringe locations, as shown in Figure 2.

The separation of two adjacent fringe peaks, i.e., FSR, is determined from the phase-matching condition Equation (5) as

(6) $$\varphi_{FSR}=\varphi_{r(m+1)}-\varphi_{rm}=2\pi .$$

When the fringe spectrum is plotted versus frequency, the corresponding FSR $f_{FSR}$ is constant for non-dispersive optical waves and is related to the phase velocity as follows:

(7) $${v_{p}=2lf}_{FSR}.$$

Equation (7) expresses the FSR as a function of the cavity distance *l* and the phase velocity *v_p_*. In FPR-based sensors, $f_{FSR}$ is determined from the measured fringe spectrum. The phase velocity $v_{p}$ or the cavity length *l* is then calculated from $f_{FSR}$ using Equation (7) if either *l* or *v_p_* is known. In practice, a mechanical measurand such as strain or pressure can change the cavity length *l* while an environmental and bio-chemical measurand such as hydrogen gas could change the refractive index of the cavity medium, which in turn could lead to a change in $f_{FSR}$ [12,31].

## 3. Airy Function of Flexural Beams with Generic Boundaries

A flexural beam is a slender structural element oscillating along its transverse direction, i.e., the direction perpendicular to its longitudinal direction. Assuming a constant cross-section and following the Euler–Bernoulli beam theory, the governing equation for the displacement *w* of a flexural beam can be derived as [21]

(8) $$E^{*}I\frac{\partial^{4}w(x,t)}{\partial x^{4}}+\rho A\frac{\partial^{2}w(x,t)}{\partial t^{2}}=0,$$

where *A*, *I*, and *ρ* stand for the cross-section area, moment of inertia, and material density of the beam, respectively. For a lossy beam, the complex Young’s modulus E^*^ is represented as E^*^ = *E*(1 + *iη*), where *η* is the mechanical loss factor. The standard frequency-domain general solution of Equation (8) is expressed as

(9) $$w_{\omega }\left(x,t\right)=\left(C_{1}e^{-i\beta x}+C_{2}e^{i\beta x}+C_{3}e^{-\beta x}+C_{4}e^{\beta x}\right)e^{i\omega t},$$

where *ω* = 2*πf* is the angular velocity and *β* = $\sqrt{\omega /a}$*= β_r_ + iβ_i_* is the complex wavenumber for a lossy beam with $a=\sqrt{E^{*}I/\rho A}$. The first two terms in Equation (9) correspond to the forward- and backward-propagating waves, respectively. The last two terms represent the non-propagating waves, also known as the evanescent waves.

**Figure 3:**
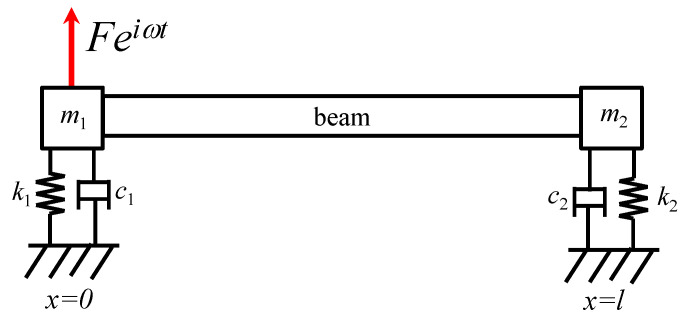
A flexural beam with both ends attached to a mass–damper–spring system. A harmonic force *Fe^iωt^* is applied at the left end.

The coefficients *C*_k_ in Equation (9) are determined from the boundary conditions. Assuming that both ends of the flexural beam are attached to a generic mass–damper–spring (MDS) system, shown in Figure 3, and a harmonic force *Fe^iωt^* is applied at the left end, the corresponding boundary conditions can be expressed as

(10) $$Left\;end:E^{*}I\frac{\partial^{2}w\left(0,t\right)}{{\partial x}^{2}}=0,E^{*}I\frac{\partial^{3}w\left(0,t\right)}{{\partial x}^{3}}-m_{1}\frac{\partial^{2}w\left(0,t\right)}{{\partial t}^{2}}-c_{1}\frac{\partial w\left(0,t\right)}{\partial t}-k_{1}w\left(0,t\right)-{Fe}^{i\omega t}=0$$

and

(11) $$Right\;end:E^{*}I\frac{\partial^{2}w\left(l,t\right)}{{\partial x}^{2}}=0,E^{*}I\frac{\partial^{3}w\left(l,t\right)}{{\partial x}^{3}}+m_{2}\frac{\partial^{2}w\left(l,t\right)}{{\partial t}^{2}}+c_{2}\frac{\partial w\left(l,t\right)}{\partial t}+k_{2}w\left(l,t\right)=0,$$

where *m*_j_*, c*_j_*,* and *k*_j_ are the mass, damping, and stiffness coefficients of the MDS systems attached to the left (*j* = 1) and right (*j* = 2) ends, respectively. Defining *A_j_ = (k_j_ − m_j_ω*^2^*) + ic_j_ω* and *B =* $E^{*}$Iβ^3^ and applying Equation (9) to the boundary conditions (10) and (11) yield

(12) $$\left[\begin{array}{cccc} B+A_{1} & -B+A_{1} & -iB+A_{1} & iB+A_{1}\\ e^{\beta l}(B+A_{2}) & e^{-\beta l}(-B+A_{2}) & e^{i\beta l}(-iB+A_{2}) & e^{-i\beta l}(iB+A_{2})\\ 1 & 1 & -1 & -1\\ e^{\beta l} & e^{-\beta l} & -e^{i\beta l} & -e^{-i\beta l} \end{array}\right]\left[\begin{array}{c} \begin{array}{c} C_{1}\\ C_{2} \end{array}\\ \begin{array}{c} C_{3}\\ C_{4} \end{array} \end{array}\right]=\left[\begin{array}{c} \begin{array}{c} F\\ 0 \end{array}\\ \begin{array}{c} 0\\ 0 \end{array} \end{array}\right].$$

Solving Equation (12) using the MATLAB 2025b symbolic solver produces a long expression for the forward-propagating wave coefficient *C*_1_, which can be re-arranged as follows:

(13) $$C_{1}=\frac{F_{3}e^{\beta l\left(1+i\right)}+F_{4}e^{-\beta l\left(1-i\right)}-2B}{F_{1}e^{\beta l\left(1+i\right)}-F_{2}e^{\beta l\left(1-i\right)}+F_{5}e^{-\beta l\left(1+i\right)}+F_{6}e^{-\beta l\left(1-i\right)}-4B^{2}i}=\frac{F_{3}+F_{4}e^{-2\beta l}-2Be^{-\beta l}e^{-\beta li}}{F_{1}-F_{2}e^{-2\beta li}+F_{5}e^{-2\beta l}e^{-2\beta li}+F_{6}e^{-2\beta l}-4B^{2}ie^{-\beta l}e^{-\beta li}},$$

where

(14) $$\begin{array}{c} F_{1}={iB}^{2}-2A_{1}A_{2}+A_{1}B\left(1-i\right)+A_{2}B\left(1-i\right),\\ F_{2}=-{iB}^{2}-2A_{1}A_{2}+A_{1}B\left(1+i\right)+A_{2}B\left(1+i\right),\\ F_{3}=\left(-2A_{2}-B+Bi\right),\\ F_{4}=\left(2A_{2}+B+Bi\right),\\ F_{5}=iB^{2}-2A_{1}A_{2}-A_{1}B\left(1-i\right)-A_{2}B\left(1-i\right),\\ F_{6}=iB^{2}+2A_{1}A_{2}+A_{1}B\left(1+i\right)+A_{2}B\left(1+i\right). \end{array}$$

The terms containing $e^{-2\beta l}$ or $e^{-\beta l}$ correspond to evanescent waves, which decay rapidly compared to the oscillatory terms containing $e^{-i2\beta l}$ and the constant terms (e.g., *F*_1_ and *F*_3_). The magnitudes of these two terms are proportional to $e^{-2\beta_{r}l}$ or $e^{-\beta_{r}l}$, respectively, where $\beta_{r}=Re(\beta )$. As such, these terms become negligible for $\beta_{r}l\gg 1$. Physically, FPR results from interference of waves “circulating” within the FPR cavity [4]. Since evanescent waves decay rapidly, they do not circulate and thus do not contribute to the FPR interference. As such, Equation (13) reduces to the following simplified form:

(15) $$C_{1}=\frac{F_{3}}{F_{1}-F_{2}e^{-i2\beta l}}.$$

Expressing the constant terms in polar forms, i.e.,

(16) $$F_{1}=F_{1}e^{i\varphi_{1}},F_{2}=F_{2}e^{i\left(-\varphi_{2}\right)},F_{3}=F_{3}e^{i\varphi_{3}}$$

and re-arranging Equation (15) leads to

(17) $$C_{1}=\left(\frac{F_{3}}{F_{1}}\right)e^{i\left({\varphi_{3}-\varphi }_{1}\right)}\left[\frac{1}{1-\left(F_{2}/F_{1}\right)e^{-2\beta_{i}l}e^{-i\left(2\beta_{r}l+\varphi_{1}+\varphi_{2}\right)}}\right].$$

By defining

(18) $$\bar{F}=\left(\frac{F_{3}}{F_{1}}\right)e^{i\left({\varphi_{3}-\varphi }_{1}\right)},\;r=\left(F_{2}/F_{1}\right)e^{-2\beta_{i}l},\;\mathrm{and}\;\varphi_{r}=2\beta_{r}l+\varphi_{1}+\varphi_{2},$$

the solution for the forward-propagating wave can be expressed as

(19) $$w_{FP}\left(x,t\right)=\frac{\bar{F}e^{-i(\beta x-\omega t)}}{1-re^{-i\varphi_{r}}}.$$

Equation (19) has the same format as the generic Airy function of optical FPRs, i.e., Equation (2). As such, a direct analogy between the flexural beam and an optical FPR is established.

Based on Equation (14), $F_{1}$ and $F_{2}$ are functions of $A_{1}$, $A_{2}$, and B. *A_j_ = (k_j_ − m_j_ω*^2^*) + ic_j_ω* is related to the mass, damping, and spring coefficients at the boundary while $B=E^{*}I\beta^{3}$ is determined by the material and geometric properties of the beam. Therefore, the ratio $F_{2}/F_{1}$ reflects the combined influence of impedance mismatch and phase change at the boundaries and the properties of the beam. The quantity $r=(F_{2}/F_{1})e^{-2\beta_{i}l}$ then represents the effective round-trip attenuation factor, including both boundary-induced reflection effects and propagation loss along the beam. For passive boundary terminations and lossy propagation, the physically admissible regime is ∣r∣≤1. The phase shift $\varphi_{r}$ includes the phase shift introduced by the round-trip wave propagation $2\beta_{r}l$ and the phase shifts at the two ends *φ*_1_ and *φ*_2_, as shown in Equation (18). When *φ*_1_ and *φ*_2_ are frequency-dependent, such as when the damping coefficient of the MDS support is not zero, the phase-matching condition of the flexural-beam FPR is

(20) $$\varphi_{rFSR}=2\beta_{rFSR}l+\varphi_{1FSR}+\varphi_{2FSR}=2\pi .$$

For free and fixed supports, *φ*_1_ and *φ*_2_ are constants, which leads to $\varphi_{1FSR}+\varphi_{2FSR}=0$. As such, the FSR of the flexural-beam FPR with these boundary conditions is

(21) $$\varphi_{rFSR}=2\beta_{rFSR}l=2\pi .$$

The fringe spectrum of the flexural-beam FPR is the spectrum of the displacement $w_{FP}\left(x,t\right)$, which can be determined from Equation (19) as follows:

(22) $$W_{FP}\left(x,f\right)=\frac{\bar{F}e^{-i\beta x}}{1-re^{-i\varphi_{r}}}.$$

Since it is identical to the FRF of the flexural beam in classical beam theory, for simplicity, the remainder of this paper refers to the fringe spectrum of the flexural-beam FPR as FRF.

From a sensing perspective, connecting the flexural-beam FRF to the FPR fringe spectrum provides an interference-based interpretation of how physical and environmental perturbations affect the resonance characteristics of flexural beams. Applying the established FPR concepts to analyze these resonant characteristics is expected to achieve advantages comparable to those of optical and microwave FPR sensors, including ultra-sensitive, robust, and reference-free measurements, as discussed in the Introduction. For example, changes in one or more of the boundary condition parameters influence the effective reflection amplitude r, which affects the fringe finesse, and the end reflection phases $\phi_{1}$ and $\phi_{2}$ that shift the resonant frequencies. However, if $\phi_{1}$ and $\phi_{2}$ are frequency-independent, Equation (21) shows that the FSRs are solely determined by $\beta_{r}$ and l. Accordingly, the FSR may serve as a reference-free spectral quantity for interpreting changes in wavenumber or effective cavity length. Moreover, the boundary condition parameters are analytically embedded in the variables $A_{1}$ and $A_{2}$. As such, impedance changes at the boundaries are directly reflected in the amplitude and phase terms of the FPR Airy function. The explicit connection between these parameters and the FPR resonant spectra enables physics-based analysis of measured FRF and FSR changes [29,30,32], rather than relying on tracking resonant frequency shifts or amplitude changes, which are commonly used in existing studies [33,34,35].

## 4. Wave Propagation in Free–Free Flexural Beam

One major advantage of mechanical FPRs is that their fringe spectra can be calculated from the Fourier transform of the displacement and thus have complex values. In contrast, the fringe spectra of optical FPRs are measured from the light intensity, having only real values. The availability of the complex fringe spectrum enables TFA to visualize wave propagation within the FPR cavity [36]. For this purpose, we use the well-known free–free flexural beam as an example. The beam has a length of *l* = 1 m, a cross-section area of *A* = 10^−6^ m^2^, a moment of inertia of *I* = 10^−13^ m^4^, and a density of *ρ* = 2715 kg/m^3^, the same as that studied in [23]. The FRF of the free–free beam at the right end, x=l, was obtained from the full solution of Equation (12), in which all unknown coefficients, *C*_i_, were determined after setting the mass, damping, and stiffness coefficients at both beam ends to zero to enforce free–free boundary conditions. A complex Young’s modulus with a small imaginary part, i.e., $E^{*}$ = *E*(1 + *iη*), with *E* = 69 GPa and *η* = 10^−3^, was used. The small loss factor *η* causes the waves to die down gradually and thus eliminates numerical artifacts associated with frequency aliasing when converting the FRF from the frequency domain to the time domain.

**Figure 4:**
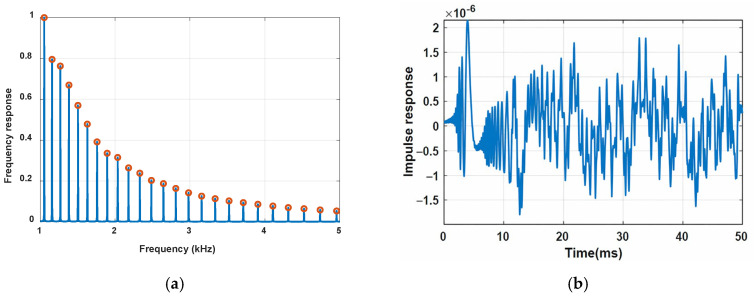
(**a**) Simulated frequency response function (FRF) of a free–free flexural beam at its right end, i.e., *x* = *l*; (**b**) impulse response converted from FRF using inverse fast Fourier transform (IFFT).

The FRF of the free–free beam was computed from the harmonic response of the beam by numerically solving Equation (12) using MATLAB at discretized frequency ranging from 1 kHz to 5 kHz with a frequency step of 1 Hz. It displays a series of sharp peaks with decaying amplitude and increasing spacing, as illustrated in Figure 4a. These peaks are called resonance peaks in classical beam theory, while they are known as the constructive interference fringes in FPR terminology. The increase in the FSR with frequency is different from that of non-dispersive FPRs, in which the FSR is a constant [23]. The impulse response of the free–free flexural beam is calculated from the inverse fast Fourier transform (IFFT) of the FRF and is shown in Figure 4b. The predominant peak at 3.9 ms corresponds to the first arrival of the flexural impulse traveling from the left end of the beam to the right end. The signals following the dominant peaks indicate wave dispersion. The dispersion effect is more pronounced as time increases, making identifying the subsequent arrivals of the reflections difficult. In other words, the multiple-round-trip propagation of the wave between the ends of the free–free flexural beam is obscured by the wave dispersion over time.

**Figure 5:**
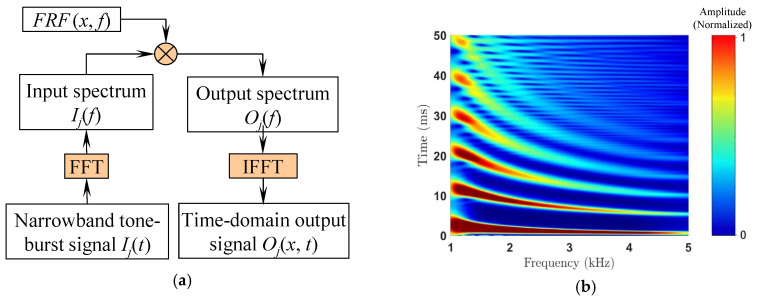
(**a**) Time–frequency analysis algorithm treating the frequency response function (FRF) as the transfer function. The subscript *j* represents the center frequency *f*_j_ of the narrowband input *I*_j_(*t*), which is swept to construct (**b**) time–frequency representation of the FRF of a free–free flexural beam revealing the Fabry–Perot nature of wave propagation.

Wave propagation within the beam can be visualized by performing TFA of the simulated FRF following the procedure shown in Figure 5a [37]. Similar to a forced vibration experiment with swept sine excitation [38], this algorithm is more physics-based than other digital signal processing algorithms such as the short-time Fourier transform (STFT). Treating the flexural beam as a linear time-invariant (LTI) system, its response to a narrowband excitation, such as a 5.5-cycle tone-burst signal, is calculated from its transfer function, i.e., the FRF. For TFA, the narrowband excitation can be digitally generated, and its spectrum can be calculated using fast Fourier transform (FFT). Multiplying the narrowband input spectrum with the FRF produces the narrowband output spectrum, from which the time-domain response of the FPR can be calculated using IFFT. Sweeping the center frequency of the narrowband excitation leads to the time–frequency representation of the FRF shown in Figure 5b. The red–yellow striation bands represent the arrival of the flexural impulse at different frequencies. The first band, arriving at approximately 4 ms, corresponds to the first arrival. The subsequent bands correspond to the arrivals of the flexural impulse after additional round trips along the beam length. Since the group velocity of the flexural beam increases with frequency, higher-frequency waves arrive earlier than the lower-frequency ones, causing the bands to curve. This dispersion effect becomes more pronounced for later-arriving bands. The separations between these bands correspond to the group velocity. At lower frequencies, the bands are further apart, indicating smaller group velocities. The reduced separation of the bands with frequency indicates increasing group velocity. The bands are relatively well defined for the first four arrivals. However, the later-arriving bands disintegrate into a series of smaller striation bands, especially at higher frequencies, making the group velocity difficult to determine. The wave-propagation characteristics of the free–free flexural beam shown in Figure 5b align well with the FPR physics described by Equation (3), confirming that a free–free beam is indeed an FPR. More generic boundary conditions, i.e., the mass, damping, and spring coefficients not being zero, influence the reflections, i.e., *F*_1_ and *F*_2_, and phase shifts, i.e., *φ*_1_ and *φ*_2_, at the boundaries (see Equation (16)). However, they do not alter the underlying FPR physics, which is the multiple reflections of the waves at the beam ends and the interference of the waves circulating inside the FPR.

It is worth noting that the procedure presented in Figure 5a is equally applicable to FRFs measured using conventional modal analysis techniques, such as exciting a beam using an impact force and measuring its responses at one location [39]. Analyzing the time–frequency representation of the FRF enables physics-based interpretation of the connection between the measured spectral variations and abnormalities such as damage, damping, or boundary condition changes [36]. Since the narrowband excitation signal is digitally generated, the time and frequency resolution of the TFA can be easily adjusted by changing the number of cycles in the excitation signal, which in turn changes its bandwidth and the TFA time and frequency resolution [37].

## 5. Relationship Between Group Velocities and Free Spectral Range

A closed-form relationship between the phase velocity and FSR is well established for non-dispersive FPR, which forms the fundamental basis for inversely determining measurands, such as pressure or temperature, from the measured FSR. Unfortunately, such a relationship has not been reported for dispersive FPRs. For dispersive waves, the group velocity, which represents the speed at which the envelope of a wave packet travels, is more relevant physically than the phase velocity [40]. It is defined as

(23) $$v_{g}=\frac{d\omega }{dk},$$

in which $k=\beta_{r}$ is the real part of the wavenumber. At a fringe peak *f_m_*, Equation (23) can be approximated as

(24) $$v_{g}\left(f_{m}\right)\approx \frac{∆\omega }{∆k}=\frac{2\pi \left(f_{m+1}-f_{m}\right)}{k_{m+1}-k_{m}}=\frac{{2\pi f}_{FSR}(f_{m})}{k_{FSR}},$$

in which

(25) $$f_{FSR}(f_{m})=f_{m+1}-f_{m}\mathrm{and}\;k_{FSR}=k_{m+1}-k_{m}.$$

Since the phase FSR $\varphi_{FSR}=2k_{FSR}l=2\pi$, plugging $k_{FSR}=\pi /l$ into Equation (24) leads to

(26) $$v_{g}\left(f_{m}\right)\approx {2lf}_{FSR}(f_{m}).$$

Equation (26) follows from a first-order finite-difference approximation of $v_{g}=\frac{d\omega }{dk}$ evaluated between two adjacent fringe peaks. By Taylor expansion,

(27) $$\frac{\omega \left(k+∆k\right)-\omega (k)}{∆k}=v_{g}\left(k\right)+{\left.\frac{1}{2}\frac{d^{2}\omega }{{dk}^{2}}\right|}_{\xi }∆k,$$

where ξ∈[k, k+∆k]. Therefore, the approximation error is first order in ∆k, with $∆k=k_{FSR}=\pi /l,$ and depends on the local curvature of the dispersion relation. The approximation is therefore more accurate when the dispersion curve varies smoothly over one fringe interval or for a long beam.

**Figure 6:**
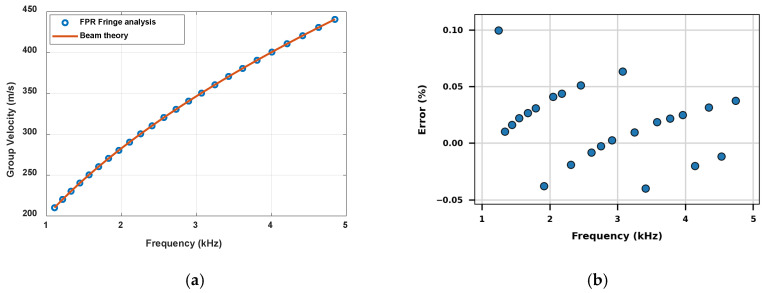
(**a**) Comparison of the dispersion curves of the flexural beam determined from the FPR fringe analysis and from classical beam theory; (**b**) percentage of error between the fringe-based and theoretical group velocities.

The dispersion curves of the flexural beam, i.e., the group velocities at different resonant frequencies, were determined from the FRF shown in Figure 4a using Equation (26) and compared with those from the classical beam theory, i.e., $v_{g}=d\omega /d\beta =2\sqrt{\omega a}$, in Figure 6a. The differences between these two dispersion curves, shown in Figure 6b, are less than 0.1%. Their excellent agreement further confirms that the FPR and classical beam theories describe the same underlying physics of wave propagation in flexural beams. The accuracy of the fringe-based group-velocity extraction depends on how precisely adjacent fringe frequencies are identified from the FRF. Therefore, the error in Figure 6 is influenced by the frequency resolution of the FRF and by the peak-detection accuracy, which could be further improved using other peak-detection techniques such as zero-crossing [41].

The closed-form relationship between the group velocity and FSR provides a straightforward interference-based framework for inversely determining the measurand from the measured FRF of dispersive flexural waveguides. For example, an external load either increases or reduces the beam length and thereby shifts the fringe peaks. However, if the group velocity of the beam remains constant, the change in the beam length can be determined from the FSR using Equation (26). Accordingly, the proposed fringe-analysis-based approach shares the same underlying interrogation principle as established Fabry–Perot sensing methods, in which the FSR is used for absolute or calibration-free cavity-length determination [5,6], and fringe-based analysis enables high-resolution measurement in a wide range of applications [7,8,9,10]. Furthermore, the authors’ group has produced convincing experimental results to support analyzing the FRF in the fringe spectrum frequency domain for reliable attenuation detection [29,30,32]. The present work therefore provides a theoretical foundation for developing waveguide resonance sensors in flexural beams.

## 6. Conclusions

This study confirms that a flexural beam is a mechanical FPR through three complementary approaches, i.e., mathematical derivation, wave-propagation analysis, and fringe-based group-velocity extraction, with direct implications for waveguide-based sensing frameworks. The FRF of a flexural beam with generic boundary conditions was formulated in the same format as the generic Airy function of optical FPRs, laying a theoretical foundation connecting flexural beams to optical FP cavities. The complex fringe spectrum of a free–free flexural beam, which is also the FRF of the beam, was analyzed in the time–frequency domain, revealing the FPR nature of wave propagation in the flexural beam. The group velocities determined from the FRF using the fringe analysis technique matched well with those from classical beam theory, further confirming the connection between flexural beams and FPRs. Although FPR theory and classical beam theory originate from different disciplines, their equivalence establishes an interference-based framework for interpreting resonant spectra in flexural beams. FPR fringe analysis and data analysis techniques, well established for optical and microwave FPRs, are equally applicable for flexural-beam FPRs. As such, this work establishes the theoretical framework for future development of dispersive mechanical waveguide resonance sensors.

## Data Availability

The data presented in this study is available on request from the corresponding author.
